# Association of Documentation of Legal Residency Status with Nonprescribed Hormone Use Among Hispanic/Latina Trans Women in San Francisco

**DOI:** 10.1089/heq.2019.0104

**Published:** 2020-06-23

**Authors:** Christopher J. Hernandez, Glenn-Milo Santos, Erin C. Wilson

**Affiliations:** ^1^ University of California, Berkeley School of Public Health, Division of Infectious Diseases and Vaccinology, Berkeley, California, USA.; ^2^ San Francisco Department of Public Health, San Francisco, California, USA.; ^3^ University of California, San Francisco, School of Nursing, California, San Francisco, USA.; ^4^ University of California, San Francisco, Department of Epidemiology & Biostatistics, San Francisco, USA.

**Keywords:** nonprescribed hormone use, undocumented status, sex-work, trans women

## Abstract

Undocumented immigrant trans Latinas face significant barriers to attaining gender-affirming health care and may use nonprescribed feminizing hormones. Without medical supervision, nonprescribed hormone use may lead to adverse health outcomes. This study aimed to determ*ine* if a history of being an undocumented immigrant was associated with nonprescribed hormone use among trans Latinas. We conducted a secondary analysis using baseline data from the 2016 Trans National study done in the San Francisco Bay Area. Two hundred five trans Latinas participated in the study, of whom 75 (37%) reported a history of being undocumented. We fitted a multivariable logistic regression model to determine whether having a history of being an undocumented immigrant was associated with nonprescribed hormone use while controlling for age, income, time living in San Francisco, history of sex work, and history of problems with accessing health care. The prevalence of nonprescribed hormone use was 55.9% among trans Latinas overall; however, for trans Latinas with a history of undocumented immigration status, the prevalence was 68%. There was a significant, independent association between nonprescribed hormone use and undocumented status (adjusted odds ratio [aOR]=3.20; 95% confidence interval [CI]=1.47–6.97). We also found that having a history of sex work was associated with nonprescribed hormone use (aOR=5.72; 95% CI=2.69–12.18). The prevalence of nonprescribed hormone use among trans Latinas was high and is associated with a history of undocumented status and sex work. These associations may indicate health care avoidance related to concerns of being criminalized due to their documentation status or source of income (i.e., sex work) among trans Latinas. These findings underscore the need to reduce barriers in gender-affirming care to increase access to medically supervised hormone use, particularly among individuals with a history of undocumented status and engaged in sex work.

## Introduction

Trans women are a diverse group of people who may have specific health needs related to their gender identity. Many trans women choose to medically transition by taking feminizing hormones.^1^ Feminizing hormones can create feminine secondary sex characteristics and halt or slow the progression of male secondary sex characteristics.^2^ Physical changes induced by feminizing hormones can do much to alleviate symptoms associated with gender dysphoria^3^, resulting in better mental health and overall increased wellness. For example, a cross-sectional study found that hormonal therapy was significantly associated with a higher quality of life among trans women who took hormonal therapy.^4^

Despite the documented benefits of feminizing hormones, trans women often face barriers to gender-affirming health care, including stigma, provider refusal to offer care, and improper clinician training.^5–7^ The stigma trans women face in health care is multilevel; it includes institutional laws and practices, personal explicit and implicit biases held by health care providers, and individual stigma that has led to health care avoidance among some trans people.^8,9^

Barriers to health care may be compounded for immigrant trans women who may have language differences, privacy concerns, and fears related to documentation status and deportation.^10,11^ Indeed, Latinas have been found to avoid health care due to their immigration status.^12^ A retrospective cohort study found that undocumented Hispanic immigrants entered care with more advanced HIV infection than documented persons.^13^ Latinas who are trans may experience similar health care avoidance due to their immigration status and/or fears related to deportation.

To meet their trans-specific health care needs, some trans women use nonmedically supervised hormone therapy, fillers, and surgeries that are not under the guidance of trained and medically licensed clinicians.^14^ A 2010 study in San Francisco found that 68.7% of trans women were on hormones; however, 49.1% of trans women in the study reported using nonprescribed hormones, with the primary reason being they were unable to access a clinician.^15^ The use of nonmedically prescribed and supervised hormones can lead to adverse health complications among trans people, especially for those on estrogen.^16,17^ Documented adverse health complications include loss of erectile function, low libido, pituitary adenoma, and venous thromboembolism.^2^ Higher rates of estrogen-mediated health complications among trans women can be reduced through clinician education and proper hormone therapy management,^18^ indicating the importance of clinical guidance for hormone therapy.

A qualitative photovoice study conducted among trans Latinas in North Carolina identified unsafe hormone use as a priority health risk.^19^ In the same study, trans Latinas reported gender-affirming health care barriers most trans women face, including few providers offering hormone therapy, stigma, and lack of access to formal health care; however, they also reported that a lack of health insurance and a paucity of bilingual health care providers prevented them from accessing prescribed hormones.^19^ As a result, many used nonprescribed hormones. Anti-immigrant sentiment is also a significant mediator of adverse health outcomes.^20,21^ The multiple levels of discrimination affecting sexual and gender minorities coupled with the legal barriers for immigrants heightens the fear in accessing health care services.^22^ Nonprescribed hormone therapy may be one of the few options available to trans Latinas for accessing gender-affirming care. However, the relationship between legal residency status and nonprescribed hormone use among trans Latinas remains underexplored.

The purpose of this study was to determine whether history of undocumented legal residence status among trans Latinas in the San Francisco Bay Area was associated with the use of nonprescribed hormones. There have been studies attempting to describe the reasons for nonprescribed hormone use among trans women overall, but little research has been done with trans Latinas who may face additional barriers due to their immigration status. There is a dearth of literature focused on the lives and health care access for undocumented trans Latinas in general and in the San Francisco Bay Area. Understanding the prevalence of and reasons why trans Latinas access nonprescribed hormones is critical to prevent adverse health outcomes and better serve this population.

## Methods

This was a secondary analysis of baseline cross-sectional data from the Trans National study of trans women living in the San Francisco Bay Area from 2016 to 2017 (*N*=629). The primary aim of the Trans National cohort study was to assess HIV incidence among trans women living in four cities around the world, and the methods are explained elsewhere.^23,24^

Respondent-Driven Sampling was used to obtain a diverse sample of hard-to-reach adult trans women from the community in San Francisco in 2016. Eligibility criteria were (1) 18 years and older, (2) resident of San Francisco by self-reporting living in San Francisco, (3) assigned male sex at birth and identified as a gender other than man (e.g., female, trans woman, woman, nonbinary, gender queer), and (4) spoke English or Spanish. After providing informed consent, participants completed an interviewer-administered computerized survey that addressed demographics, behavioral history, clinical history, gender identity, access to health care, and enrollment in insurance.

All statistical analyses were done using STATA version 16 (College Station, TX). In this study, we restricted our analysis to trans women participants who identified as Hispanic/Latina. Our analysis focused on two research areas; (1) do trans Latinas use hormones, nonprescribed hormones and do they have health insurance access?, and (2) is having a history of being undocumented among trans Latinas associated with nonprescribed hormone use? Descriptive statistics were provided on self- and laboratory-verified health indicators (i.e., HIV, Hepatitis C, mental health), health care use, health insurance and resilience for trans Latinas by documentation status compared among groups U.S. Born, documented immigrant, and undocumented immigrant participants.

The outcome of interest was measured with the question “Have you ever used nonprescribed hormones?.” The main independent variable, documentation status, was measured with the question “Have you had a history of being undocumented?.” Current documentation status was intentionally not asked to reduce concern participants may have about disclosing documentation status. Theoretical correlates with nonprescribed hormone use, sex work, and problems accessing health care, were assessed through “Have you ever performed sex work?” and “have you experienced difficulty in accessing healthcare?.” The questions measured a historical outcome and exposures and were not necessarily representative of current status.

Hepatitis C virus (HCV) antibodies were detected using Oraquick^®^ HCV Rapid Antibody Test. HIV screening was done using INSTI^®^HIV-1/HIV-2Rapid Antibody Test. Gender identity was assessed by the question “What is your gender identity?.” Ten options were provided, including “prefer not to state.” Mental health history was assessed by asking “Have you been diagnosed with any of the following mental health issues?,” with the following options provided; depression, anxiety, post-traumatic stress disorder (PTSD), and other.

Current insurance enrollment and use of health care facilities were assessed. Insurance access was assessed by the question “What kind of health insurance or coverage do you currently have?” and had the following options; No insurance, HMO/Private, Medicaid/Medi-Cal, Medicare, Healthy San Francisco, Tri-Care, Veteran's Affairs, and other. Access to gender-affirming care facilities was assessed by “Did you use any of the following services in 2015?,” with the following options; TransThrive/API Wellness Center, Transgender Surgery Access Program at SFDPH Transgender Health Services, LGBT Center/Transgender Employment Services, Lyon Martin Health Services, and Tri-City Health Center. Difficulties accessing health care and a history of sex work are known barriers to health care and can affect access to gender-affirming care of nonprescribed hormone.^25,26^

A bivariate logistic regression analysis was conducted between nonprescribed hormone use and demographic characteristics, health indicators, health care use, gender-affirming health care use, health insurance, history of sex work, by documentation status compared among groups U.S. born, documented immigrant, and undocumented immigrant participants.

To build a multivariable model, the method described by Hosmer and Lemeshow was employed, in which predictors that were statistically significant at the bivariate level (*p*<0.20) were included in the larger multivariable model.^27^ A stepwise backward procedure was used with potential demographic confounders retained in the model. The likelihood ratio test was used to confirm that the nested models fit the data as well or better than the larger models. We ran goodness-of-fit test on the model and found that a continuous age variable and ordered categorical variable for time living in San Francisco provided the best fit. Data with missing values were excluded from the analysis. The study received Institutional Review Board approval from the University of California, San Francisco's Human Research Protection Program (IRB # 15–17775.).

## Results

A total of 205 trans women identified as Latina and were included in this analysis. Ninety-eight (48.0%) reported being born in the United States, and 107 (52.2%) reported being immigrants. Overall, trans Latinas mostly identified as transgender (53.2%), and 38.1% identified as female (Table 1). The most common sexual orientation reported was being straight (62.0%), and trans Latinas born in the United States had a higher proportion of reporting being genderqueer, androgynous, or another sexual orientation.

**Table 1.** Characteristics of Trans Latinas in the Trans National Study, San Francisco, CA; 2016–2017 (*N*=205)

Trans-Latina demographics	U.S. born (n=98)	No history of undocumented status (n=30)	History of undocumented status (n=77)	Total
	N (%)	N (%)	N (%)	N (%)
Education				
Less than high school	12 (12.2)	6 (20.0)	4 (5.2)	22 (10.3)
High school diploma/GED	20 (20.41)	14 (46.7)	37 (48.1)	71 (34.6)
Some college/technical	31 (31.6)	4 (13.3)	19 (24.7)	54 (26.3)
College degree/beyond	35 (35.7)	6 (20.0)	17 (22.1)	58 (28.3)
	98	30	77	205
Time in the United States				
1–4 years	—	9 (30.0)	8 (10.7)	17 (16.2)
5–10 years	—	0 (0.0)	10 (13.3)	10 (9.5)
11–15	—	1 (3.3)	19 (25.3)	20 (19.1)
16–20	—	6 (20.0)	12 (16.0)	18 (17.1)
21+	—	14 (46.7)	26 (34.7)	40 (38.1)
		30	75	105
Time in SF				
0–2 years	23 (24.0)	9 (30.0)	15 (20.0)	47 (23.4)
3–6 years	12 (12.5)	3 (10.0)	5 (6.7)	20 (10.0)
7–10	8 (8.3)	1 (3.3)	18 (24.0)	18 (13.4)
11+	53 (55.2)	17 (56.7)	37 (49.3)	37 (53.2)
	96	30	75	201
Age				
18–19	0 (0.0)	1 (3.3)	0 (0.0)	1 (0.5)
20–24	16 (16.5)	2 (6.7)	7 (9.3)	25 (12.4)
25–29	21 (21.7)	4 (13.3)	6 (8.0)	31 (15.4)
30–34	10 (10.2)	3 (10.0)	10 (13.3)	22 (10.9)
35–39	13 (13.4)	2 (6.7)	13 (17.3)	28 (13.9)
40–44	10 (10.3)	5 (16.7)	17 (22.7)	32 (15.8)
45–49	8 (8.3)	2 (6.7)	7 (9.3)	17 (8.4)
50–59	14 (14.4)	7 (23.3)	14 (18.0)	33 (16.3)
60+	6 (6.2)	4 (13.3)	3 (4.0)	13 (6.4)
	98	30	77	205
Extremely low income for SF				
Above poverty level	31 (31.3)	4 (13.3)	9 (12.2)	44 (21.7)
At or below Poverty level	66 (68.6)	26 (86.7)	67 (88.2)	159 (78.3)
	97	30	76	203
Gender identification				
Female	43 (43.9)	10 (33.3)	25 (32.5)	78 (38.1)
Transgender	42 (42.9)	20 (66.7)	47 (61.0)	109 (53.2)
Androgynous	2 (2.0)	0 (0.0)	1 (1.3)	3 (1.5)
Genderqueer	7 (7.1)	0 (0.0)	1 (1.3)	8 (4.0)
Questioning	0 (0.0)	0 (0.0)	1 (1.3)	1 (0.5)
Other	4 (4.1)	0 (0.0)	2 (2.6)	6 (3.0)
	98	30	77	205
Sexual orientation				
Straight	46 (47.4)	20 (66.7)	61 (79.2)	127 (61.9)
Gay/lesbian	5 (5.2)	3 (10.0)	4 (5.3)	12 (5.9)
Bisexual	16 (16.5)	5 (16.7)	4 (5.3)	25 (12.4)
Pansexual	12 (12.4)	0 (0.0)	0 (0.0)	12 (5.9)
Queer	11 (11.3)	1 (3.3)	3 (4.0)	15 (7.4)
Questioning	3 (3.1)	0 (0.0)	0 (0.0)	3 (1.5)
Other	5 (5.1)	1 (3.3)	5 (6.7)	11 (5.3)
	97	30	100	205
Income: (% of group)				
Job	48 (49.9)	8 (26.7)	29 (38.7)	85 (42.1)
GA, food stamps	28 (28.8)	6 (20.0)	24 (31.2)	58 (28.3)
SSI	32 (32.7)	8 (26.7)	7 (9.1)	47 (22.9)
Disability	11 (11.2)	6 (20.0)	3 (4.0)	20 (9.8)
Unemployment	2 (2.06)	0 (0.0)	1 (1.3)	3 (1.5)
Main partners income	11 (10.2)	1 (3.3)	7 (9.1)	19 (9.3)
Family's income	20 (20.4)	4 (13.3)	8 (10.4)	32 (15.6)
Sex work	28 (28.6)	4 (13.3)	10 (13.0)	42 (20.5)
Drug dealing	2 (2.0)	0 (0.0)	0 (0.0)	2 (1.0)
Alimony/child support	1 (1.0)	0 (0.0)	0 (0.0)	1 (0.5)
Student loans	5 (5.21)	0 (0.0)	0 (0.0)	5 (2.4)
HIV status (lab result)				
Positive	28 (28.6)	11 (36.7)	19 (24.7)	58 (28.3)
Total tests run	98	30	77	205
HCV status (lab result)				
Positive	21 (29.2)	2 (10.0)	8 (15.4)	31 (21.4)
Total tests run	72	20	53	145
Mental health				
Depression	61 (62.9)	14 (46.7)	32 (42.7)	107 (52.9)
PTSD	32 (32.9)	8 (26.7)	17 (22.7)	57 (28.2)
Anxiety	59 (60.2)	13 (43.3)	36 (46.8)	108 (52.7)
Health insurance				
HMO (%yes in that group)	18 (18.4)	3 (10.0)	5 (6.5)	26 (12.7)
Medi-Cal/medicaid	55 (56.1)	21 (70.0)	38 (49.4)	114 (56.0)
Medicare	5 (5.1)	0 (0.0)	1 (1.3)	6 (3.0)
Veterans administration	0 (0.0)	0 (0.0)	0 (0.0)	0 (0.0)
Tricare	1 (1.0)	0 (0.0)	0 (0.0)	1 (0.5)
Healthy SF	15 (15.31)	9 (30.0)	30 (39.0)	54 (26.3)
Other	7 (7.1)	0 (0.0)	5 (6.5)	12 (5.9)
No insurance	7 (7.2)	0 (0.0)	5 (6.7)	12 (5.9)
Services accessed	(% from 97)	(% from 30)	(% from 75)	Total
Trans thrive/API Wellness Center	26 (26.5)	11 (36.7)	15 (19.5)	52 (25.4)
Transgender surgery access SFDPH	8 (8.2)	2 (6.7)	9 (12.0)	19 (9.3)
LGBT Center/transgender employment	19 (19.4)	9 (30.0)	9 (11.7)	37 (18.1)
Lyon Martin Health Center Services	11 (11.2)	3 (10.0)	6 (8.0)	20 (9.8)
Tri-City Health Center	5 (5.1)	0 (0.0)	3 (4.0)	8 (4.0)
Other services	20 (20.4)	9 (30.0)	20 (25.3)	49 (23.8)
No services	22 (22.5)	4 (13.3)	16 (21.0)	42 (20.5)
Gender-affirming surgeries				
Laser hair removal	32 (32.7)	9 (30.0)	22 (29.0)	—
Penectomy	1 (1.0)	1 (3.3)	(0.0)	
Orchiectomy	9 (9.3)	3 (10.0)	3 (4.0)	—
Vaginoplasty	7 (7.1)	4 (13.3)	4 (5.2)	
Breast implants	11 (11.2)	6 (20.0)	27 (35.1)	—
Facial feminization	3 (3.1)	3 (10.0)	9 (11.7)	
Dermal filler	3 (3.1)	0 (0.0)	(0.0)	—
Electrolysis	6 (8.7)	0 (0.0)	2 (4.3)	
Voice surgery	1 (1.5)	0 (0.0)	0 (00.0)	—
Tracheal shave	2 (3.0)	0 (0.0)	1 (2.1)	
Fat transfer	0 (0.0)	0 (0.0)	1 (2.1)	
Voice therapy	2 (2.9)	0 (0.0)	0 (0.0)	—
No surgery done	50 (51.6)	13 (43.3)	38 (50.7)	
History of sex work				
No	26 (27.0)	12 (40.0)	27 (35.0)	65 (31.9)
Yes	71 (73.0)	18 (60.0)	50 (64.9)	139 (68.1)
	97	30	77	
Currently on hormones?				
No	31 (32.0)	8 (26.7)	28 (36.4)	67 (32.7)
Yes	67 (68.4)	22 (73.3)	49 (64.0)	138 (67.3)
	98	30	77	205
Hormones covered by insurance? Among those taking hormones				
No	9 (13.3)	(0.00)	3 (6.3)	12 (8.8)
Yes	58 (86.6)	22 (100)	45 (93.8)	125 (91.2)
	67	22	48	137
Taken nonprescribed hormones?				
No	50 (51.0)	16 (53.3)	24 (31.0)	90 (44.5)
Yes	48 (49.0)	14 (46.7)	53 (69.0)	115 (56.9)
	98	30	77 (100)	205

HCV, hepatitis C virus; PTSD, post-traumatic stress disorder.

The most common forms of income were employment for those born in the United States (49.0%) and sex work (28.6%). Those not born in the United States with no history of being undocumented reported income from jobs (26.7%), general assistance and/or food stamps (20%), social security income/SSI (26.7%), and disability (20%). Those not born in the United States with a history of being undocumented reported income from a job (37.7%), general assistance and/or food stamps (31.2%), and sex work (13.0%). One hundred fifty-nine (78.3%) trans Latinas reported an income at or below the poverty level for San Francisco. Seventy-seven (37.6%) reported a history of being undocumented. Among U.S.-born trans Latinas, 71 (73.2%) reported a history of doing sex work as a source of income. Among immigrants with and without a history of documentation, 18 (60.0%) and 50 (65.0%) reported a history of engaging in sex work, respectively.

Fifty-eight (28.3%) of trans Latinas tested positive for HIV. Those trans Latinas not born in the United States but without a history of being undocumented had the highest HIV prevalence (37.7%). Thirty-one (21.4%) of trans Latinas tested antibody positive for the HCV. U.S.-born trans Latinas had the highest HCV prevalence of 29.2%, while 10% of immigrants without a history of being undocumented had HCV, as did 15% of immigrants with a history of being undocumented. Mental health conditions were highest for U.S.-born trans Latinas. Among U.S.-born trans Latinas, 63.3% reported depression, 32.7% reported PTSD, and 60.2% reported anxiety. Among immigrants with documentation, 46.7% reported depression, 26.7% reported PTSD, and 44.8% reported anxiety. Among immigrants with a history of being undocumented, 44.2% reported symptoms of depression, 23.4% reported PTSD, and 46.8% reported anxiety.

In this study, 193 (93.0%) trans Latinas reported having health insurance coverage. The two most common reported health insurance plans were Medical/Medicaid (55.6%) and the health access program Healthy San Francisco (26.3%). Seventy-two trans Latina immigrants (93.5%) with a history of an undocumented status had some form of health insurance access. Most (67.3%) trans Latina participants reported currently using hormone therapy, and 115 of the participants in this study (56.1%) had a history of nonprescribed hormone use. Among trans Latinas not born in the United States with a history of being undocumented, 49 (64.0%) were currently on hormones, and 53 (68.8%) reported having used nonprescribed hormones at some point.

In the multivariable analyses, nonprescribed hormone use was significant and independently associated with greater odds of a history of undocumented status (adjusted odds ratio [aOR]=3.37, 95% confidence interval [CI]=1.55–7.33) and a history of sex work (aOR=5.41, 95% CI=2.56–11.41), while controlling for age, poverty status, time living in San Francisco, education, and reporting having problems getting health care coverage (Table 2).

**Table 2.** Left: Bivariate Associations with Non-Prescribed Hormone Use Among Trans Latinas. Right: Multivariable Associations With Non-Prescribed Hormone Use Among Trans Latinas. Trans National Study San Francisco, CA; 2016–2017 (*N*=205)

Bivariate model		Multivariable model		
Characteristic	OR (95% CI)	p	aOR (95% CI)	p
Age	1.02 (0.99–1.04)	0.091	1.02 (0.99–1.06)	0.106
Poverty	1.63 (0.83–3.21)	0.159	1.51 (0.66–3.48)	0.330
U.S. born	—	—	Reference	—
Documented immigration status	0.91 (0.40–2.06)	0.824	0.78 (0.29–2.06)	0.610
History of undocumented immigration status	2.30 (1.23–4.29)	0.009	3.37 (1.55–7.33)	0.002
Gender identification				
Female	—	—	—	
Transwoman	0.87 (0.48–1.57)	0.644	—	
Queer/additional	0.33 (0.11–0.97)	0.044	—	
Time living in San Francisco				
0–2 years	—	—	Reference	—
3–6 years	1.08 (0.38–3.07)	0.892	1.25 (0.37–4.20)	0.720
7–10 years	0.95 (0.37–2.44)	0.912	0.33 (0.10–1.07)	0.064
11+ years	1.26 (0.63–2.51)	0.512	0.74 (0.30–1.79)	0.500
Enrolled in healthy SF	2.57 (1.31–5.04)	0.006	—	—
Education				
Less than high school	—		Reference	—
HS diploma/GED	0.48 (0.23–0.99)	0.047	0.46 (0.14–1.59)	0.177
Some college/technical	0.91 (0.44–1.87)	0.796	1.21 (0.37–3.98)	0.226
College degree or beyond	0.32 (0.12–0.86)	0.024	1.06 (0.32–3.46)	0.752
History of sex work	4.24 (2.26–7.94)	0.000	5.41 (2.56–11.41)	0.000
HIV result (+)	0.91 (0.75–1.10)	0.311	—	—
HCV result (+)	0.88 (0.61–1.28)	0.518	—	—
Problems getting health care	1.55 (0.63–3.84)	0.344	1.86 (0.66–5.23)	0.237
Breast augmentation	2.51 (1.21–5.21)	0.014	—	—
Facial feminization	12.33 (1.59–95.70)	0.016	—	—
Ever used fillers	6.38 (2.55–15.96)	0.000	—	—
Housing instability	2.4 (1.30–4.42)	0.005	—	—
Depression	1.52 (0.87–2.65)	0.136	—	—
Anxiety	1.66 (0.96–2.91)	0.071	—	—
PTSD	1.92 (1.01–3.62)	0.045	—	—

aOR, adjusted odds ratio; CI, confidence interval.

## Discussion

Nonprescribed hormone use was associated with having a history of undocumented immigration status for trans Latinas in the San Francisco Bay Area. In this sample, a large proportion of trans Latinas overall used nonprescribed hormones, however trans Latinas with a history of being undocumented were significantly more likely to report nonprescribed hormone use relative to U.S.-born immigrants or trans Latina immigrants who did not report a history of being undocumented (68% for undocumented trans Latinas, vs. 49.5% and 46.7% for U.S.-born and trans Latinas with documented immigration status, respectively).

The finding that having a history of being undocumented was associated with accessing hormones outside the health care system is consistent with studies that being undocumented presents a significant barrier to gender-affirming health care.^11^ Trans Latina immigrants with a history of being undocumented in our sample may have experienced greater racism and stigma, and faced more barriers to economic opportunities, which may have restricted their access to gender-affirming care due to lack of health insurance and health care avoidance.^19^ Trans Latinas with a history of being undocumented may have also been reluctant to engage with the health care system for fear of deportation, and instead turned to other resources to access nonprescribed hormones.^19^ The use of entitlement programs like Medicaid can delay or negate the process for obtaining legal residency in the United States,^28^ and trans Latinas may forgo gender-affirming care access from entitlement programs to reduce risk of jeopardizing their path toward becoming a documented immigrant.

We also found that nonprescribed hormone use was associated with sex work among trans Latinas. Almost two thirds of participants reported a history of doing sex work. When comparing between U.S. born, documented immigrants, and immigrants with a history of being undocumented, trans Latinas born in the United States had the highest proportion of a history of sex work and sex work as a current source of income, speaking to the importance of sex work as a source of income regardless of immigration status. For trans women, the higher rates of unemployment, poverty, and homelessness than the general population contribute to engagement in sex work as a means of income.^29^ Sex work can create a number of structural barriers to health care, and thus hormone access. Due to a lack of formal employment, trans Latinas who do sex work may face structural barriers to health care as they do not have access to employer-sponsored insurance. The stigma associated with sex work has also been documented as a significant barrier to health care and presents a challenge to reaching individuals who engage in sex work.^30,31^ Trans Latinas who do sex work may also be fearful of criminalization if their health care provider finds out their occupation. Trans Latinas who engage in sex work may also not be available for medical visits as clinics are primarily open during business hours.

The use of nonprescribed hormone use among trans Latinas in our study presents a cautionary tale for San Francisco and other places where trans-specific services exist in that availability does not mean access or utilization for all trans people. San Francisco and neighboring counties have trans-specific services, including surgery access, and health insurance access. In fact, there are numerous trans-specific services in the San Francisco Bay Area, as demonstrated in our data showing that most had used trans-specific services in San Francisco and 90% of trans Latinas had health care insurance or were enrolled in the city's health care access program. Also, city policies are in place to ensure immigrants can access health care. In 1989, San Francisco passed the “City and County of Refuge,” and in 2007, San Francisco launched Healthy San Francisco, a health access program for its most marginalized communities, including those living in poverty and undocumented people.^32,33^

Despite these policies and services, trans Latinas, especially those with a history of being undocumented, are using nonprescribed hormones, suggesting that while these policies are necessary and helpful, they may not be sufficient to ensure maximum uptake of medically supervised gender-affirming care. The use of nonprescribed hormones among trans Latinas with a history of undocumented status and those who report sex work may be an indication that this vulnerable population is avoiding engagement in health care services out of fear of criminalization of their source of income (i.e., sex work) and their documentation status.

The primary limitation of this study is that these data are cross-sectional and reporting on past experiences of using nonprescribed hormones and prior undocumented status. Thus, the lack of information on the temporal sequence between our exposure and outcome limits our ability to conclude definitively that nonprescribed hormone use is predicted by sex work and being undocumented among trans Latinas. Data were also self-reported and social desirability bias, especially concerning documentation status and sex work, could lead to underreporting. Additionally, all participants were trans Latinas living in San Francisco, which could limit the generalizability of our findings. Due to the lack of census data on trans women anywhere in the world, it is difficult to determine how representative our study population is to the general trans Latina community. However, this study is valuable in that it highlights the vulnerabilities trans Latinas face while in San Francisco, informing areas of need that we need to address.

Despite limitations, the association between factors that could be criminalized and use of nonprescribed hormones among trans Latinas observed in this study gives us important insight into the potential barriers to care faced by some of the most vulnerable members of the trans women population in San Francisco. The use of nonprescribed hormones poses a considerable health risk for trans Latinas who may face heightened risk for pulmonary issues, blood clots, and other life-threatening health complications that are preventable with regular medical monitoring.^34,35^ Trans Latinas, like all trans people who desire feminizing hormones, should have a right to safe and quality health care free from the fear of criminalization and deportation. Future research is needed to determine why and how the existing services are not meeting the needs for hormones and how to build trust in the system among those most vulnerable to criminalization and deportation in the trans Latina community.

## Abbreviations Used

aOR: adjusted odds ratio
CI: confidence interval
HCV: hepatitis C virus
PTSD: post-traumatic stress disorder
